# Cytotoxic T Cell Expression of Leukocyte-Associated Immunoglobulin-Like Receptor-1 (LAIR-1) in Viral Hepatitis C-Mediated Hepatocellular Carcinoma

**DOI:** 10.3390/ijms232012541

**Published:** 2022-10-19

**Authors:** Reham Hammad, Reda Badr Aglan, Shaymaa A. Mohammed, Eman Abu-elnasr Awad, Marwa A. Elsaid, Hanan M. Bedair, Seham K. Khirala, Mohamed A Selim, Asmaa A. Abo Elqasem, Areej Rushdi, Mohamed Ali, Omaima I. Abo-Elkheir, Eman F. Sanad, Nadia M. Hamdy

**Affiliations:** 1 Clinical Pathology Department, Faculty of Medicine (for Girls), Al-Azhar University, Nasr City, Cairo 11884, Egypt; 2 Hepatology and Gastroenterology Department, National Liver Institute, Menoufia University, Menoufia 35211, Egypt; 3 Internal Medicine Department, Faculty of Medicine (for Girls), Al-Azhar University, Nasr City, Cairo 11884, Egypt; 4 Clinical Pathology Department, National Liver Institute, Menoufia University, Menoufia 32511, Egypt; 5 Microbiology and Immunology Department, Faculty of Medicine for Girls, Al-Azhar University, Nasr City, Cairo 11884, Egypt; 6 Botany and Microbiology Department, Faculty of Science (for Boys), Al-Azhar University, Nasr City, Cairo 11884, Egypt; 7 Immunology, Zoology and Entomology Department, Faculty of Science, Al-Azhar University (for Girls), Nasr City, Cairo 11884, Egypt; 8 Clinical Pharmacy Department, Faculty of Pharmacy, Ain Shams University, Abassia, Cairo 11566, Egypt; 9 Community Medicine and Public Health, Faculty of Medicine, Al-Azhar University, Nasr City, Cairo 11884, Egypt; 10 Biochemistry Department, Faculty of Pharmacy, Ain Shams University, Abbasia, Cairo 11566, Egypt

**Keywords:** T cytotoxic cells, leukocyte-associated immunoglobulin-like receptor-1, LAIR-1, hepatitis C virusgenotype4, HCV G4, hepatocellular carcinoma, cirrhosis, immune inhibitory checkpoints, inflammation, prognosis, insulin resistance

## Abstract

Virus-related hepatocellular carcinoma (HCC) pathogenesis involves liver inflammation, therefore, despite successful treatment, hepatitis C virus (HCV) may progress to HCC from initiated liver cirrhosis. Cytotoxic T cells (Tcs) are known to be involved in HCV-related cirrhotic complications and HCC pathogenesis. The inhibitory checkpoint leukocyte-associated immunoglobulin-like receptor-1 (LAIR-1) is expressed on Tcs. Therefore, we aimed to determine whether the Tc expression level of LAIR-1 is associated with HCC progression and to evaluate LAIR-1 expression as a noninvasive biomarker for HCC progression in the context of liver cirrhosis related to HCV genotype 4 (G4) in Egyptian patients’ peripheral venous blood liquid biopsy. A total of 64 patients with HCC and 37 patients with liver cirrhosis were enrolled in this case-controlled study, and their LAIR-1 expression on Tc related to the progression of liver cirrhosis was examined and compared to that of the apparently healthy control group (*n* = 20). LAIR-1 expression was analyzed using flow cytometry. Results: The HCC group had significantly higher LAIR-1 expression on Tc and percentage of Tc positive for LAIR-1 (LAIR-1+Tc%) than the HCV G4-related liver cirrhosis group. LAIR-1+Tc% was correlated with the HCC surrogate tumor marker AFP (*r* = 0.367, *p* = 0.001) and insulin resistance and inflammation prognostic ratios/indices. A receiver operating characteristic (ROC) curve revealed that adding LAIR-1+Tc% to AFP can distinguish HCC transformation in the Egyptian patients’ cohort. Upregulated LAIR-1 expression on Tc could be a potential screening noninvasive molecular marker for chronic inflammatory HCV G4 related liver cirrhosis. Moreover, LAIR-1 expression on Tc may be one of the players involved in the progression of liver cirrhosis to HCC.

## 1. Introduction

Background. Liver cancer, particularly hepatocellular carcinoma (HCC), is the third leading cause of cancer-related deaths worldwide, accounting for approximately 80% of primary liver cancers [1]. Despite significant advancements in HCC care, liver cancer diagnosis, and surveillance, HCC-related deaths remain unacceptably high [2]. Most HCC cases happen in the milieu of chronic inflammatory disease(s), with cirrhosis being the strongest risk factor for HCC [3]. HCV infection is among the main risk factors for HCC development [4]. However, hepatitis C virus (HCV) risk has considerably decreased due to direct-acting antiviral drugs [5]. Nevertheless, even after successful HCV treatment, patients with cirrhosis are still believed to have a higher risk of HCC [6]. Additionally, 90% of HCC cases develop from persistent chronic HCV inflammation [4] despite successful HCV treatment.

HCC is considered an immunogenic tumor; therefore, the best therapy would be immune therapeutics [7]. Cytotoxic T cells (Tcs) have central role in the HCC pathogenesis and control of HCV infection. Moreover, Tcs can recognize tumoral/infected cells and subsequently destroy them [8].

Regarding HCC, various studies have reported shifting the immune response toward the anti-tumor direction. Tc cell restoration, together with modulation of the negative co-stimulatory signaling molecules expressed on these cells, could have an anti-tumoral impact [9,10]. A careful balance between activating and inhibitory signals determines the immune system’s proper response to damage. Maintaining this equilibrium is greatly aided by inhibitory receptors. One of the immune inhibitory receptors is leukocyte-associated immunoglobulin-like receptor-1 (LAIR-1), an immunological checkpoint widely expressed on immune cells to deliver inhibitory signals [11].

Following a literature search and research gap discussion mentioned in publications, we have chosen LAIR-1/CD305 as the target of our study. LAIR-1 is a transmembrane type I glycoprotein of two tyrosine-based inhibitory motifs immunoreceptor in the cytoplasm and an external Ig-like domain C2-type [12,13]. LAIR-1 expression has been described in cells of the immune system [11,14]. It has been observed that LAIR-1 expression is normally elevated in immune cells; however, tumor cells hijack this immune regulatory system to avoid the “anti-cancer immune response” [15]. In the tumor microenvironment, the decrease in the immune activity, along with a decline in T-cell function, is caused by LAIR-1 binding to its collagen or other ligands [13]. The role of LAIR-1 in chronic inflammatory processes, such as rheumatoid arthritis [16] and systemic lupus erythematosus [17], suggest that LAIR-1, as an inhibitory receptor, may contribute to the pathophysiology of post-chronic inflammation, including post-HCV. Indeed, it is crucial to comprehend innate immune signaling and the immune regulators governing different signaling pathways to anticipate the liver cirrhosis prognosis and/or progression from liver cirrhosis to HCC post-HCV G4.

We hypothesize that the level of the immune inhibitory receptor LAIR-1 expression on circulating Tc is associated with HCV-related liver cirrhosis progression to HCC. Unraveling the molecular events in HCC blood liquid biopsy and underlying Tc cell function may provide a guide for identifying potential drug targets.

Aim(s) and Objective(s). First, to understand if the level of LAIR-1 expression on Tc is associated with liver cirrhosis progression to HCC. Second, to evaluate LAIR-1 expression as a sensitive, noninvasive prognostic biomarker in the peripheral blood liquid biopsy of Egyptian patients with HCC. Moreover, to discover whether Tc contributes to liver cirrhosis progression to HCC in Egyptian patients with HCV genotype 4 (G4) after successful treatment through LAIR-1 involvement, as LAIR-1 resistance.

## 2. Results

### 2.1. Participants’ Demographic and Clinical Characteristics

This study included 101 patients with HCC who were categorized into HCV G4 mediated HCC (*n* = 64) and HCV G4-related liver cirrhosis (*n* = 37) groups and compared to 20 apparently healthy control volunteers (Table 1). This case-controlled study revealed no significant differences regarding age, gender, or kidney function (serum creatinine results).

Comparison between the clinical characteristics of the studied HCV G4-related HCC and liver cirrhosis cases and the apparently healthy control group is shown in Table 2.

Body mass index (BMI), serum insulin and insulin resistance, and platelet-to-lymphocyte ratio (PLR), an inflammation prognostic ratio, decreased, whereas TAG/HDL-C and GGT-to-lymphocyte ratio (GLR), an HCC prognostic ratio, increased (Table 2). Moreover, the increased levels of liver function and AFP were all significant between the two groups and the healthy control group (Table 2). The HCC group had a significantly increased percentage of total lymphocytes than the liver cirrhosis group, whereas the percentage of Tc% showed no significant difference when compared within groups. Additionally, the comparison of LAIR-1 expression revealed a significant increase in the post-HCV G4 group compared with the control group. LAIR-1 mean fluorescence intensity (MFI) on Tc and LAIR-1+Tc% were 38.5 (range, 29.3–49.2) and 75.20 (range, 59.3–87.0) in the patients’ groups, whereas their levels in the healthy control group were 22.0 (range, 17.5–31.2) and 13.6 (range, 8.3–19.0), respectively.

### 2.2. Participants’ Pathological Characteristics

The pathological characteristics of the study participants are presented in Table 3. Ascites, CHILD score for liver affection severity, lymph node (LN), and lung findings were all significantly different between the liver cirrhotic and HCC groups.

### 2.3. LAIR-1 Expression Levels in Patients Groups

**Figure 1:**
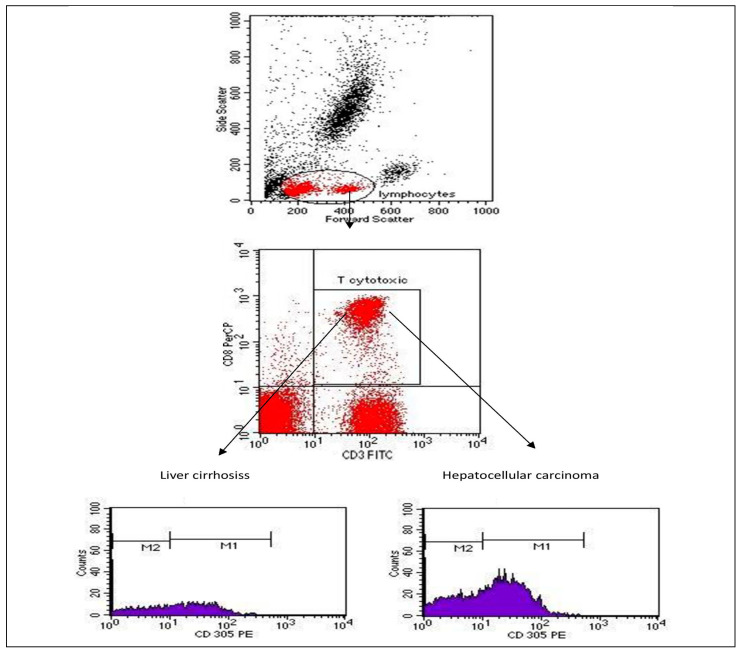
Percentage of Tc expressing LAIR-1/CD305 and LAIR-1 MFI on single histogram from CD3+CD8+ co-expressing population for HCC group (bottom right) and liver cirrhosis (bottom left) obtained by FC initial gating by typical forward and sideways scatter on mature lymphocytes expected area. (N.B. The far-right population are monocytes with higher side scatter and bigger size, noted by the forward scatter, that is why they are excluded in lymphocytes gating). [MFI; mean fluorescence intensity of positive population detected on area under M1 marker.].

A stratified analysis of disease susceptibility and LAIR-1 expression level is presented in Table 4. A summary of the comparison between groups regarding all laboratory analyses is also shown in Table 4. No significant difference was noted between groups in gender or BMI. However, significant difference was noted between patients with HCC and patients with liver cirrhosis regarding serum insulin, presence of insulin resistance and liver function ALT, AST, GGT, AFP, serum albumin, GLR, TAG/HDL-C, and LAIR-1 expression. Flow cytometry (FC) results are presented in Figure 1.

### 2.4. Correlation between the LAIR-1 Expression Level and Patients with HCC and Liver Cirrhosis

Spearman correlation included all patients (*n* = 101) and revealed that both the frequency of LAIR-1+Tc and LAIR-1 MFI on Tc were positively correlated with AFP (Table 5). LAIR-1 MFI on Tc was positively correlated with insulin resistance and GLR. Moreover, LAIR-1+Tc was positively correlated with liver size and PV patency.

LAIR-1+Tc was significantly positively correlated with LAIR-1 MFI on Tc (*r* = 0.734, *p* < 0.001).

AFP levels were positively correlated with inflammation markers (PLR and GLR) (*r* = 0.201, *p* = 0.040 and *r* = 0.214, *p* = 0.032, respectively).

### 2.5. ROC Curve for the Discriminative Ability of LAIR-1+Tc% and LAIR-1 MFI on Tc to Differentiate HCV-Related HCC from Liver Cirrhosis

**Figure 2:**
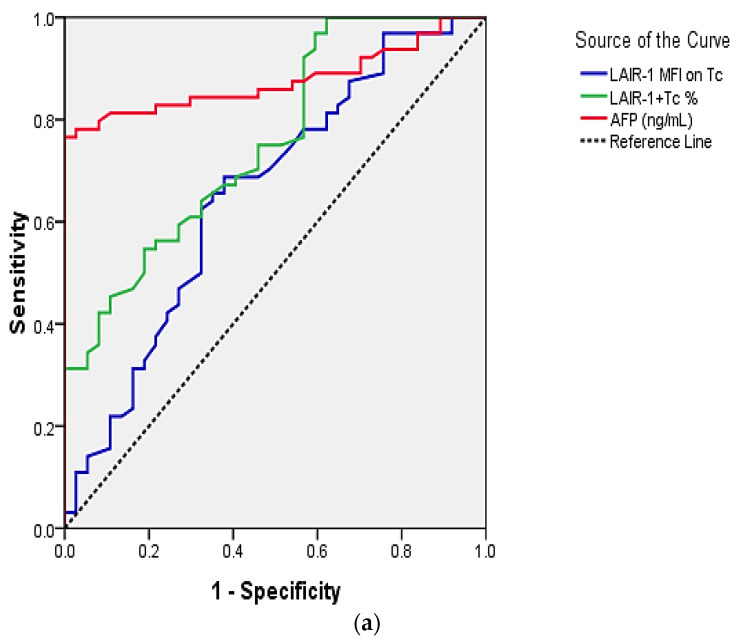

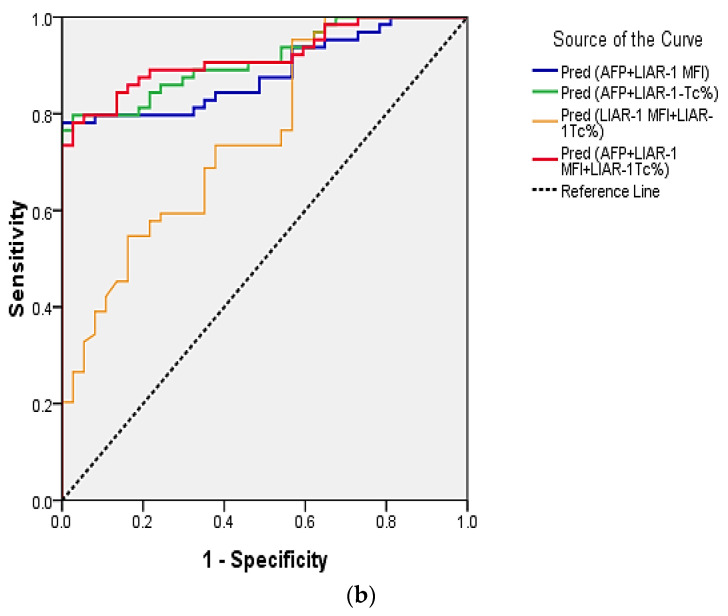
ROC curve for the discriminative ability of (**a**) LAIR-1+Tc%, LAIR-1 MFI on Tc or AFP to differentiate HCC from liver cirrhosis in comparison to (**b**) different combination of variables.

LAIR-1+Tc% at a cutoff value of ≥73.6 and an AUC of 0.756 can distinguish HCV G4 related HCC progression from HCV G4 related liver cirrhosis with 67.2% sensitivity and 62.2% specificity (*p* < 0.001). LAIR-1 MFI on Tc at a cutoff value of >34.5 and an AUC of 0.651 had the same sensitivity and specificity (*p* = 0.012). AFP at a cutoff value of ≥10.2 ng/mL and an AUC of 0.876 had 82.2% sensitivity and 75.7% specificity (*p* < 0.001) (Table 6, Figure 2).

### 2.6. In Silico Biology

#### 2.6.1. Gene Location

**Figure 3:**
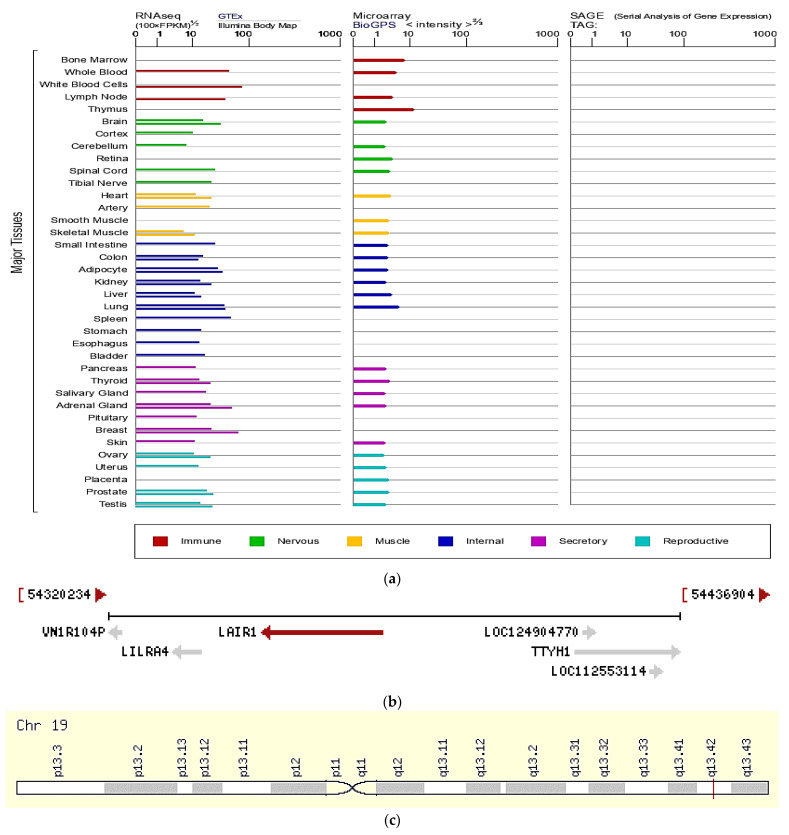
*LAIR-1* gene characteristics (**a**) mRNA expression in normal human tissues from GTEx, Illumina, BioGPS, SAGE for *LAIR-1* gene https://www.genecards.org/cgi-bin/carddisp.pl?gene=LAIR1&keywords=LAIR-1, (**b**) genomic context https://www.ncbi.nlm.nih.gov/gene/3903 (accessed on 29 July 2022). (**c**) Cytogenetic band: genomic location: bands according to Ensembl, locations according to GeneLoc, latest assembly: chr19:54,351,384-54,376,088 (GRCh38/hg38), Size: 24,705 bases, Orientation: Minus strand https://www.genecards.org/cgi-bin/carddisp.pl?gene=LAIR1&keywords=LAIR-1.

LAIR-1 gene located in chromosome 19, exon 13. External Ids for LAIR-1 Gene; HGNC: 6477, NCBI Entrez Gene: 3903, Ensembl: ENSG00000167613, OMIM^®^: 602992, UniProtKB/Swiss-Prot: Q6GTX8.

https://www.genecards.org/Search/Keyword?queryString=LAIR-1 (accessed on 29 July 2022) as presented in Figure 3 addressing *LAIR-1* gene characteristics, according to https://www.genecards.org/cgi-bin/carddisp.pl?gene=LAIR1&keywords=LAIR-1, https://www.ensembl.org/Homo_sapiens/Gene/Summary?db=core;g=ENSG00000167613;r=19:54351384-54370558 (accessed on 29 July 2022).

#### 2.6.2. Functional Enrichments

**Figure 4:**
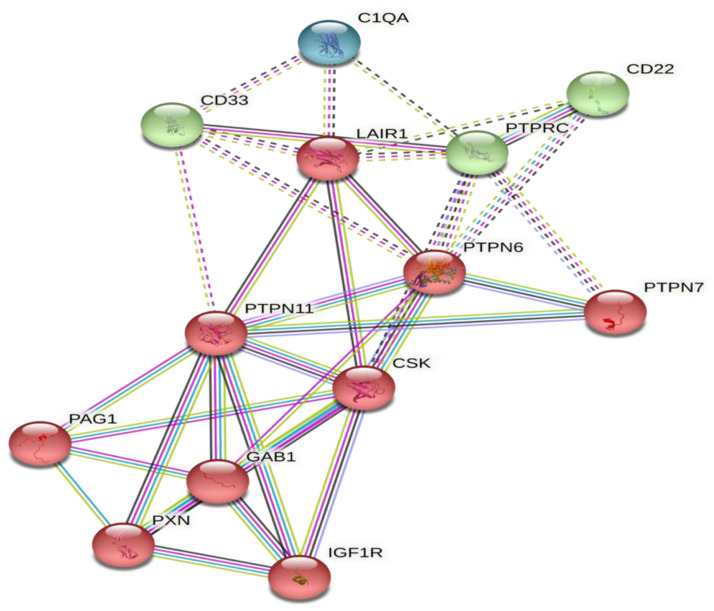
Top STRING interaction network for *LAIR1* gene preview https://string-db.org/cgi/network?taskId=bZ25wTH8KOIM&sessionId=bOdQ8mhP3zbB (accessed on 29 July 2022).

In the current work functional enrichment (Figure 4) obtained via the local network cluster (STRING) mixed constitutive signaling using https://string-db.org/cgi/network?taskId=bZ25wTH8KOIM&sessionId=bOdQ8mhP3zbB (accessed on 29 July 2022).

Where the reactome pathways are cytokine signaling in the immune system and adaptive immune system, the biological processes are cell activation, regulation of cytokine production, and regulation of cytokine-mediated signaling pathways. The molecular functions are protein tyrosine kinase and phosphatase binding and insulin receptor binding.

## 3. Discussion

The pathogenesis of HCC in Egypt mostly involves HCV-related liver inflammation, and if this occurs after successful complete HCV G4 treatment, clinical exploration is required. Liver cirrhosis is an intermediate process during the pathogenesis of HCC [18]. Therefore, understanding the molecular and cellular events underlying liver cirrhosis’ progression to HCC is essential for identifying potential therapeutic targets.

A significant factor during HCC development is an inflamed and cirrhotic liver with significant immune infiltration caused by HCV G4 infection. With few available treatments, HCC is one of the main causes of cancer-related deaths globally [19] and nationally [18]. In our patients’ cohort, the following were the clinical interrelated risk factors for HCC development: progression of cirrhosis disease to cancer; male gender; older age; diabetes; obesity with hyperinsulinemia, insulin resistance, and dyslipidemia; inflammation prognostic ratios and indices (TAG/HDL-C and GLR); and a history of HCV infection post-treatment.

The immune system recognizes tumor cells as non-self cells via Tc; therefore, the immune response is controlled by inhibitory receptors to stop autolysis. One of the inhibitory receptors on immune cells is the cytotoxic T-lymphocyte–associated antigen 4, previously measured in HCV G4-related HCC, as well as its single nucleotide polymorphism in Egyptian patients with HCV G4 [20].

Moreover, ligands for inhibitory receptors related to the immune system are constitutively expressed on healthy cells, although they can be lost in malignant cells [21]. The loss of the inhibitory checks on tumor cells will allow activating signals to predominate, which may be involved in liver cirrhosis progression to HCC HCV G4 infection or post-treatment.

The collagen-binding inhibitory receptor, *LAIR-1* gene, is encoded on chromosome 19 by the leukocyte receptor complex [22]. *LAIR-1* gene produces an inhibitory receptor that is located on peripheral blood cells as T cells. The *LAIR-1* gene belongs to the leukocyte-associated inhibitory receptor family. The leukocyte receptor cluster contains at least 29 genes encoding leukocyte-expressed receptors of the immunoglobulin superfamily domain (Figure 3b). The *LAIR-1* gene-encoded protein may cause cell death in myeloid leukemias [23] and has been identified as an anchor for tyrosine phosphatase [24].

LAIR-1 is expressed on immune cells, including CD3+CD8+ cytotoxic T cells, in the tumor microenvironment and circulation, inhibiting immune cell activities [25,26] and explaining liver cirrhosis progression to HCC.

The current study was undertaken to understand the role of Tc cells LAIR-1 level expression in primary liver cancer from HCV G4.

Based on the STRING network reactome pathways illustrated in Figure 4 cytokine signaling in the adaptive immune system, the functions of molecular LAIR-1 are protein tyrosine kinase and phosphatase binding and insulin-like growth factor receptor-related binding. In our study, the serum insulin level was significantly increased in the patients’ group (*n* = 101) compared with that in the apparently healthy control group (*n* = 20). Furthermore, diabetes, insulin resistance, and its prognostic markers in the cirrhotic group (*n* = 37) were significantly increased compared with those in the healthy control (*n* = 20) and HCC (*n* = 64) groups. This suggests that as with primary liver cancer, HCC mostly affects individuals with cirrhosis from an underlying chronic liver inflammatory disease, including HCV-related [1] as in our case.

LAIR-1 serves as an inhibitory receptor for T cells on a permanent basis, with a tyrosine phosphorylation component to activate PTPN6 and PTPN11 phosphatases. However, without the aid of phosphatases, LAIR-1 reduces anti-inflammatory cytokine IL-2 and the interferon IFN-gamma production while increasing the release of transforming growth factor beta in CD4+ T cells (per the STRING pathway). Barnabei et al. in 2021 found LAIR-1 to inhibit the nuclear translocation of nuclear factor kappa-B cell p65 subunit, phosphorylation of the inhibitory kappa-B alpha in myeloid leukemia cell lines, and proliferation inhibition and induction of apoptosis in these cells [27].

The accumulation of fatty acids, presented in our current study, that resulted as a consequence to the increased serum TAG, obesity, and dyslipidemia with diabetes, leading to generalized inflammation and triggering a pro-inflammatory cytokine, are presented clinically as significant prognostic inflammatory indices, such as TAG/HDL-C ratio and GLR. This leads to the nonalcoholic steatohepatitis component of the current patients’ cohort, a step toward liver fibrosis that precedes cirrhosis. This is consistent with a Korean population-based study showing an altered lipid metabolism being linked to HCC development with TAG (mg/dL) significantly increased in the HCC group [28].

In the current study, we aimed to examine whether LAIR-1 influences HCC susceptibility by performing a case-controlled study in a population of Egyptian patients, evaluating the association between HCV G4 post-treatment and liver cirrhosis risk or cirrhosis progression to HCC. Moreover, we aimed to determine if LAIR-1 could predict HCC prognosis with an odds ratio and 95% confidence interval under credible statistical models.

Moreover, to prove whether measuring and/or quantifying Tc LAIR-1 expression can serve as a sensitive, noninvasive prognostic molecular marker in post-HCV G4 infection-treated Egyptian patients’ peripheral blood liquid biopsy, we explored if the level of Tc expression of LAIR-1 is associated with liver cirrhosis progression compared with HCV G4-related HCC.

Tc% showed no significant difference when compared between groups, whereas the % of Tc positive for the immunoinhibitory LAIR-1 (LAIR-1+Tc%) was significantly increased in the HCC group compared with that in the liver cirrhosis group (*p* < 0.001). Regarding the expression of LAIR-1% on Tc +cells and LAIR-1 MFI on Tc, it was significantly increased in the HCC group compared with that in the liver cirrhosis group (*p* = 0.012). These findings were consistent with those of Ma et al. who showed that Tc cells are essential for anti-tumor immunity [29]. Wu et al. reported that LAIR-1 over expression in HCC tissues was significantly associated with worse overall survival [30]. Regarding LAIR-1 cross-linking with its ligand, it inhibits the cytotoxic activity of CD8+ T cells and T-cell receptor/CD3 complex signaling [31], worsening the overall survival; therefore, currently, we are working on collecting the patients overall survival (years) and LAIR-1 ligand measurement in a complementary manuscript (future prospective).

Martínez-Esparza et al. reported that blood monocytes exhibited higher LAIR-1 expression levels in cirrhotic patients and emphasized that liver cirrhosis is characterized by a progressive replacement of the functional hepatic architecture by nonfunctional fibrotic tissue, which is rich in the immunoinhibitory LAIR-1 ligand, collagen deposition [11]. Progression toward liver cirrhosis is caused by a dysregulation of immune regulatory mechanisms that govern the balance between activation/homeostasis of the immune system in case of chronic viral infections [32]. In our study, cirrhotic liver lesions had a significantly higher (*p* < 0.001) LAIR-1+Tc expression level than focal liver lesions, multiple liver lesions (Table 4), and portal vein patency. Moreover, the cases with ascites had higher LAIR-1 MFI on Tc expression level than those without ascites (*p* = 0.027); additionally, the cases with marked ascites had higher LAIR-1 MFI on Tc expression level than those with minimal ascites (*p* = 0.020). However, larger liver size and LN involvement showed a higher trend in LAIR-1 expression than N0 cases, suggesting LAIR-1 collusion during liver cirrhosis and HCC. This is in addition to the correlation of either LAIR-1+Tc or LAIR-1 MFI on Tc expression % with the tumor marker AFP (*r* = 0.367, *p* < 0.001 and *r* = 0.213, *p* = 0.033, respectively), which points out the diagnostic utility of LAIR-1 expression in HCC transformation post-HCV treatment, being measured together with AFP, proved more, by better sensitivity and specificity %, when both are plotted by the ROC curve (Figure 2 and Table 6).

Despite no significant correlation being reported between LAIR-1+Tc% or LAIR-1 MFI on Tc and lipid profile (as illustrated in Table 5), the HDL-C level was significantly decreased in the patients’ group and stepwise in both post-HCV groups, with an increased serum total cholesterol and TAG (dyslipidemia documented in Table 1 and Table 2). It is hypothesized that a link may be found between LAIR-1 expression and HDL-C during HCC development post-HCV treatment as a previous Mendelian meta-analysis reported that a 1-mg/dL reduction in the HDL-C level was associated with a 14% increased overall cancer risk [33]. Tosi et al. previously reported the scavenger receptor class B type on LAIR-1 ligand complement C1q, an HDL-C receptor, enhances the uptake of cholesteryl esters, leading to a decrease in serum HDL-C levels [34]. During liver cirrhosis progression to HCC post-HCV infection treatment, LAIR-1 overexpression is associated with Tc exhaustion, inflammation progression, and fatty acid build-up [34,35]; moreover, cholesterol crystals increase due to dysfunctional clearance [36] in the HCC microenvironment [37].

According to the ROC curves, LAIR-1+Tc%, at a cutoff value of ≥73.6 and an AUC of 0.756 can distinguish HCV-related HCC transformation from HCV-related liver cirrhosis, with 67.2% sensitivity and 62.2% specificity (*p* < 0.001), whereas LAIR-1 MFI had 67.2% sensitivity and 62.2% specificity at a cutoff value of >34.5 and an AUC of 0.651 (*p* = 0.012). AFP at a cutoff value of ≥10.2 and an AUC of 0.876 had 82.2% sensitivity and 75.7% specificity (*p* < 0.001) (Table 6, Figure 2).

This implies the clinical significance of utilizing either expression % of LAIR-1+Tc and/or LAIR-1 MFI on Tc mainly with AFP for better diagnosis, with acceptable sensitivity and specificity. Therefore, % of LAIR-1+Tc and/or LAIR-1 MFI on Tc could be a good diagnostic choice in AFP-negative HCC cases [38], a recommendation worthy of further investigation and proof in the clinical practice setting.

**Limitations.** (i) Flaws should be acknowledged, including the small sample size, lead-time bias, and selection bias. (ii) The capacity of the molecular biomarker assay refining is limited by the absence of comparable formalin-fixed paraffin-embedded tissue specimens to complement the liquid biopsy samples. Therefore, it is recommended that LAIR-1 and its ligand expression in liver tissue biopsy are performed in another future study.

**Sustainability Plan.** One ongoing study by our team addresses the role of LAIR-1 SNPs variant haplotype in the HCC Egyptian patients’ cohort. Therefore, the sustainability plan aims at continuing the team oncology research work, addressing the role of several tumor immune-related checkpoint effectors/down-stream target genes/proteins, addressing in the STRING pathway (Figure 4) in primary and/or metastatic HCC diagnosis and/or prognosis, and unraveling their exact role in post-treated HCV G4 HCC tumorigenesis.

## 4. Materials and Methods

### 4.1. Sample Size and Power Study

Based on the previous study by Gu et al., 2021 [39], the sample size estimation was performed using the G power* sample size online calculator https://riskcalc.org/samplesize/# (accessed on 10 November 2021) depending on a two-sided significance level of 0.05 and power (1-beta) of 0.95. According to Gu et al., who presented their results as a graph chart, the expression mean for each group was extracted using a graph reader tool http://www.graphreader.com/ (accessed on 10 November 2021), and standard deviations (SD) was calculated online. According to Gu et al., the gray zone group population standard deviation was (5), expected means 74, 80, and the large effect size (1.2). Therefore, calculated using the online calculator, our current study group sample size was 40 patients and 12 control participants. These sample sizes rejected the null hypothesis that the population means of the studied groups are equal with a probability (power) of 0.9.

### 4.2. Study Design

Case-controlled study.

### 4.3. Study Participants

**Healthy control participants.** A random selection of apparently healthy volunteers, not suffering from any disease or taking any medication was performed. A total of 20 control participants with normal kidney and liver functions and without any clinical or laboratory evidence of steatosis or cirrhosis were included. They were recruited during routine checkup examinations for themselves or their relatives, with ages ranging from 55.5 to 60 years (16 males, 4 females). **Patients’ groups.** This study enrolled 64 HCV G4-related HCC patients recruited from the Hepatology and Gastroenterology Department, National Liver Institute, Menoufia University, with ages ranging from 57.3 to 67 years (50 males and 14 females). The HCV G4-related HCC group included 37 patients with HCV G4-related liver cirrhosis recruited from the Faculty of Medicine, Alzahraa Hospital, Al-Azhar University, with ages ranging from 54.5 to 66 years (28 males and 9 females). Full history was collected and recorded for all study participants (*n* = 101). Blood samples were collected at the time of diagnosis for those who met the inclusion criteria and signed the informed consent. Blood samples were rejected for those who met the exclusion criteria. **Patients’ inclusion criteria** were adults over 18 years old with confirmed pathological examination of newly diagnosed HCC of no specific type post-HCV G4 (confirmed by serology). The **exclusion criteria** were patients with blood disorders, HBV (as determined by serology), schistosomiasis, HIV, thyroid dysfunction, inflammatory diseases, and cardiovascular disorders. Furthermore, patients with HCC who were not associated with HCV were excluded. Participants receiving any chemotherapy or radiotherapy or whom had undergone a gastrointestinal surgical procedure, had any cancer other than HCC, and those with neuronal diseases, uterine diseases, kidney diseases, and prolonged use of corticosteroids or sex hormones were excluded. Finally, patients with incomplete data or incomplete histopathology diagnosis report were excluded. **Patients’ clinical and pathological features.** Patients’ tumor clinical assessment was performed at the Pathology Unit, Faculty of Medicine. An abdominal computed tomography (CT) scan was performed by an expert who was blinded to the study. CT data were CT scan liver size and mass number from 1 to 4, incidence of cirrhosis with heterogeneous mass, or focal or multiple lesions and the portal vein, whether occluded or thrombosed [40]. The International Ascites Club [41] classifies the severity classification of ascites as mild ascites (grade 1), ascites only detectable by ultrasound; moderate ascites (grade 2), moderate abdominal distention; and massive ascites (grade 3), marked large abdominal distention. Regarding the severity of liver disease, the Child-Pugh-Turcotte scoring was attempted; A least severe, B moderately severe, C most severe. BCLC staging for HCC were 0 very early stage, A early, B intermediate, C advanced, and D terminal stage.

LN involvement with enlargement incidence either as no (N0) or present (N1) was recorded from patients’ data. HCV was the G4 endemic in Egypt, confirmed by serology. HCV G4 was confirmed in patients with liver cirrhosis more than 6 months ago. According to recent standards, HCC diagnosis was histologically verified or based on specified imaging criteria [42], and only early diagnosed, treatment-naïve patients with HCC were included.

### 4.4. Blood Samples

Six milliliters of peripheral venous blood were withdrawn from each participant under strict sterile conditions following standard biosecurity and international safety procedures and divided into two aliquots. Blood was withdrawn at the time patients were first diagnosed clinically with HCC and before any medical therapy or surgical intervention. The first 3 mL of blood was placed into EDTA anticoagulant vacutainers for complete blood count using Sysmex KX-21 (the automated haematology analyser KX-21 manufactured by Sysmex Corporation, Kobe, Japan) and FC assay. The rest of the blood was transferred into a polymer serum gel separator tube with a clot activator (Kremsmünster, Upper Austria, Greiner Bio-One GmbH, Australia) left for 15 min at room temperature (24 °C) to clot, followed by 10-min centrifugation at 10,000× *g* at 4 °C. Sera obtained were aliquoted into Eppendorf tubes and stored at −80 °C until biochemical assessment.

### 4.5. Research Setting

Clinical Pathology Department, Faculty of Medicine, Al-Azhar University, Cairo and the Advanced Biochemistry Research Lab, Biochemistry Dept., Faculty of Pharmacy, Ain Shams University, Cairo, Abassia.

#### 4.5.1. Biochemical Testing

**Routine biochemical testing. Liver function tests**: alanine transaminase (ALT), aspartate transaminase (AST), gamma-glutamyl transferase (GGT), alkaline phosphatase (ALP), total and direct bilirubin, and serum albumin. **Lipid profile measurements**: triacylglycerol (TAG), total cholesterol (TC), and high-density lipoprotein-cholesterol (HDL-C). Finally, serum creatinine as a **kidney function indicator**. Serum glucose determination. All were performed using Cobas Integra 400 Plus, Roche Diagnostics, Germany. **Serum alpha-fetoprotein** (AFP) determination was performed using the electrochemiluminescence immunoassay (ECLIA) using Cobas 6000 (e601 module), Roche Diagnostics, Germany. **INR** coagulation assay using an automated coagulation analyzer (STA Compact Max, Asnières sur Seine Cedex, Stago, France).

Serum insulin assay was performed using enzyme immunoassay for the quantitative determination of human serum insulin concentrations [43]. Based on an enzyme-linked immunosorbent test (ELISA) in solid phase, using a HyPrep automated ELISA system (Hyperion Inc., Miami, FL, USA). The test sample’s color intensity is directly inversely related to its insulin concentration. The normal insulin adult range level is 0–25 mU/L.

#### 4.5.2. Ratios and Indices

Weight in kilograms and height in meters was recorded for all participants for BMI (in kg/m^2^) calculation according to; https://www.nhlbi.nih.gov/health/educational/lose_wt/BMI/bmicalc.htm (accessed on 23 July 2022) with normal weight = 18.5–24.9 kg/m^2^, overweight = 25–29.9 kg/m^2^, and obesity = BMI of 30 kg/m^2^ or greater morbid obesity.

**TAG/HDL-C ratio** with a cutoff value of more than the healthy control group average was set diagnostic for insulin resistance (IR) [44].

**PLR** as a systematic inflammation biomarker and immune response-related indicator, superior to the neutrophil-to-lymphocyte ratio for HCV infections and HCC for correlation with disease severity [45].

GLR as prognostic for HCC with size < 5 cm [46].

**Insulin Resistance** is considered positive in obese, diabetic, and dyslipidemic patients, and those with insulin levels of 18 mU/mL or more after glucose/meal, with disturbed PLR [47,48].

#### 4.5.3. FC Assay

FC was performed for Tc and lymphocyte % quantification and LAIR-1 expression determination. FC was conducted using the four-color FACSCalibur (Biosciences Becton, Dickinson and Company, San Jose, California, CA, USA). One tube was prepared for each patient, using 50 µg of fresh peripheral venous blood sample after adjustment of peripheral blood mononuclear cells cell count (1 × 10^6^) in each sample. Blood was incubated with 5 µg of each fluorochrome-conjugated antibody anti-human IgG, fluorescein isothiocyanate (FITC) conjugated anti-human CD3 (BD Biosciences, San Jose, California, CA, USA. Cat. No. 555332), phycoerythrin (PE)-conjugated anti-human CD LAIR-1 (BD Biosciences, San Jose, California, CA, USA. cat. no. 550811, lot no. 5329747), and peridinin–chlorophyll–protein complex (PerCP)-conjugated anti-human CD8 (Analysis, Thermo Fisher, Waltham, MA, USA, cat. no. MA1-19793) for identification of Tc cells expressing LAIR-1. The compensation setting was established before acquiring samples using color-calibrated beads (BD, Biosciences, San Jose, California, CA, USA, lot no. 5093879).

**Gating strategy (antibody testing strategy):** Initial gating by typical forward and sideway scatter on mature lymphocytes expected area was performed, and Tc cells were then evaluated as the percentage of total lymphocytes, according to the surface marker expression as CD3^+^CD8^+^. Subsequently, the percentage of Tc expressing LAIR-1 and LAIR-1 MFI were detected on single histogram from CD3^+^CD8^+^ co-expressing population. The MFI of positive population was detected in the area under the M1 marker (Figure 1).

### 4.6. Statistical Analysis

Data collected were coded and analyzed using the Statistical Package for Social Science software (SPSS, Version 17, Chicago, IL, USA). Qualitative data were presented as frequencies (*n*) and percentages (%). Test for data normality was performed using the Shapiro–Wilk calculator, wherein normally distributed variables were expressed as means + SDs and analyzed using two samples independent Students’ *t*-test or ANOVA for comparison of two or more groups, respectively. Data were presented as medians (interquartile ranges as first to third quartiles or 25th–75th quartiles) if not normally distributed; subsequently, the Mann–Whitney (U) or Kruskal–Wallis (H) test was performed to compare any two or more independent groups, respectively. Kruskal–Wallis post hoc was conducted to determine which pairs of groups significantly differed. Student’s *t*-test and Chi-square χ2 test were used to compare quantitative and qualitative, normally distributed variables between the patients and control groups, respectively. Spearman’s *rho* correlation test was used to assess the association between quantitative nonparametric variables. Point-biserial correlations were used to determine the correlation between parameters when one of them was a dichotomous variable. ROC curve was performed to detect the best cutoff, sensitivities (SNs), and specificities (SPs), with an area under the curve (AUC) calculated range from 0 to 1. The higher the AUC, the better the parameter in classifying the outcomes correctly. ROC curve calculated values would provide an idea of how well the discriminating ability of LAIR-1+Tc% and/or LAIR-1 MFI on Tc to differentiate post-treated HCV G4 HCC cases from liver cirrhosis cases. The level of significance was set at *p*-value < 0.05 and confidence level or interval at 95% and 5%, respectively.

## 5. Conclusions

LAIR-1 expression is significantly upregulated in circulating T cytotoxic cells in HCV G4-related HCC when compared with liver cirrhosis. LAIR-1 expression on Tc comes second to AFP sensitivity and specificity as a potential screening molecular marker for HCC related to HCV G4. However, the sensitivity of LAIR-1 expression as a noninvasive molecular marker in liquid biopsy is accepted for the detection of HCC progression from liver cirrhosis HCV G4-related infection, which opens the door for better screening of AFP-negative HCC transformation. The LAIR-1 expression on Tc is associated with insulin resistance and inflammation in the context of HCC prognosis HCV G4-related, confirming the LAIR-1 resistance component.

## Figures and Tables

**Table 1:** Study participants’ demographic and clinical characteristics in all patients’ group (*n* = 101) compared to the apparently healthy control participants group (*n* = 20).

	Groups, *n*		
Characteristics (Unit)	Patients, 101	Healthy Control, 20	*p* Value
Gender (M/F)	78/23	16/4	NS
Age (years)	61.0(55.0–67.0)	58.0(55.5–60.0)	NS
BMI (Kg/m^2^)	29.3(27.2–32.0)	27.2(26.6–27.8)	<0.001 *
D.M (Yes/No)	43/58	0/20	<0.001 *
s.Insulin(mIU/L)	20.0(10.13–33.0)	8.1(6.3–10.0)	<0.001 *
Insulin resistance (Yes/No)	62/39	0/20	<0.001*
s.Albumin (mg/dl)	3.2(2.5–3.7)	3.25(3.1–3.5)	NS
AST (U/L)	58.0(43.0–81.0)	30.5(24.2–38.7)	<0.001 *
ALT (U/L)	42.0(29.0–55.5)	27.0(23.0–36.5)	0.003 *
Total Bilirubin(mg/dl)	1.6(1.0–3.4)	0.85(0.52–1.0)	<0.001 *
Direct Bilirubin(mg/dl)	0.90(0.4–2.1)	0.30(0.20–0.4)	<0.001 *
ALP (U/L)	112.0(78.0–151.0)	51.0(39.5–61.0)	<0.001 *
GGT (U/L)	54.0(40.0–70.0)	19.0(17.2–23.7)	<0.001 *
Total Cholesterol (mg/dl)	149.0(111.5–192.0)	155.5(151.2–161.7)	NS
TAG (mg/dl)	122.0(88.0–189.5)	114.5(98.2–122.7)	NS
HDL-C (mg/dl)	34.0(27.0–41.5)	46.5(42.2–50.7)	<0.001 *
TAG/HDL-C ratio	3.8(2.4–6.1)	2.3(2.1–2.7)	<0.001 *
TLC (/mm^3^)	7.5(4.5–11.8)	8.3(7.4–9.6)	NS
Hgb (gm/dl)	11.0(8.9–12.6)	13.0(12.0–13.8)	<0.001 *
PLTs (×10^9^/L)	126.0(90.0–231.0)	244.5(223.0–276.0)	<0.001 *
INR	1.3(1.2–1.6)	1.1(1.0–1.3)	<0.001 *
Lymphocyte %	24.8(19.0–32.0)	23.5(20.6–29.7)	NS
PLR	81.4(52.5–123.1)	117.7(93.1–172.0)	0.005 *
AFP (ng/mL)	15.3(5.8–163.0)	4.5(2.8–6.6)	<0.001 *
Tc%	19.0(13.8–23.0)	17.4(11.9–28.0)	NS
LAIR-1 MFI on Tc	38.5(29.3–49.2)	22.0(17.5–31.2)	<0.001 *
LAIR-1+Tc %	75.20(59.3–87.0)	13.6(8.3–19.0)	<0.001 *
GLR	28.7(17.6–45.5)	9.1(7.6–11.0)	<0.001

Data are median (interquartile range (1st–3rd quartile)), statistics were computed using SPSS software, Mann–Whitney test (non-parametric data), * statistical significance *p*-value < 0.05, NS, non-significant. [ALT, alanine aminotransferase; AST, aspartate aminotransferase, ALP, Alkaline phosphatase; AFP, alpha feto protein, BMI, Body mass index; HDL, high-density lipoprotein; INR, international normalized ratio; GGT, gamma glutamyl transferase; GLR, GGT-to-lymphocytes ratio; LAIR-1, Leukocyte-associated immunoglobulin-like receptor-1; LC, liver cirrhosis; PLT, platelet; Tc. T cytotoxic; TLC, total leukocytic count.].

**Table 2:** Study participants’ demographic and clinical characteristics (unit) in HCV G4-related HCC group (*n* = 64), HCV G4-related liver cirrhosis group (*n* = 37) compared to each other and to the apparently healthy control participants group (*n* = 20).

Groups, *n* Characteristics (Unit)	HCC, 64	Liver Cirrhosis, 37	Healthy Control, 20	*Significance*		
				*p1*	*p2*	*p3*
Gender (M/F)	50/14	28/9	16/4	NS	NS	NS
Age (years)	62.0(57.3–67.0)	60.0(54.5–66.0)	58.0(55.5–60.0)	NS	0.006 *	NS
BMI (K.gm/m^2^)	29.0(27.0–31.0)	29.4(28.0–33.8)	27.2(26.6–27.8)	NS	NS	0.005 *
D.M (Yes/No)	25/39	18/19	0/20	NS	0.001 *	<0.001 *
s.Insulin (mIU/L)	22.3(12.9–35.2)	14.4(5.4–24.9)	8.1(6.3–10.0)	0.002 *	<0.001 *	0.048 *
Insulin resistance (Yes/No)	47/17	15/22	0/20	0.001 *	0.001 *	0.001 *
s.Albumin (mg/dl)	3.4(2.9–3.7)	2.6(2.1–3.7)	3.2(3.1–3.5)	0.004 *	NS	0.016 *
AST (U/L)	63.0(52.0–94.2)	38.0(23.0–68.0)	30.5(24.2–38.7)	<0.001	<0.001	NS
ALT (U/L)	45.0(34.3–61.7)	30.0(19.0–40.0)	27.0(23.0–36.5)	<0.001 *	<0.001 *	NS
Total Bilirubin(mg/dl)	1.4(1.0–2.3)	2.3(1.0–6.45)	0.85(0.52–1.0)	NS	<0.001 *	<0.001 *
Direct Bilirubin(mg/dl)	0.80(0.40–1.5)	1.1(0.40–3.3)	0.30(0.20–0.40)	NS	<0.001 *	<0.001 *
ALP (U/L)	112.0(83.3–169.3)	102.0(73.0–139.5)	51.0(39.5–61.0)	NS	<0.001 *	<0.001 *
GGT (U/L)	56.0 (45.0–76.0)	39.0(25.0–63.0)	19.0(17.2–23.7)	<0.001	<0.001 *	<0.001 *
Total Cholesterol (mg/dl)	154.5(117.2–212.0)	130.0(105.0–171.0)	155.5(151.2–161.7)	NS	NS	NS
TAG (mg/dl)	134.5 (94.5–205.7)	105.0(65.0–140.5)	114.5(98.2–122.7)	0.006 *	NS	NS
HDL-C (mg/dl)	34.0(26.0–39.7)	35.0(29.0–43.4)	46.5(42.2–50.7)	NS	<0.001 *	<0.001 *
TAG/HDL-C ratio	4.1(2.8–6.8)	3.1(2.0–5.8)	2.3(2.2–2.7)	0.02 *	<0.001 *	NS
TLC × 10^3^/mm^3^	7.3(4.2–11.1)	7.8(4.9–12.1)	8.3(7.4–9.6)	NS	NS	NS
Hgb (gm/dl)	11.3(9.9–12.8)	9.1(8.0–12.3)	13.0(12.0–13.8)	0.003 *	0.001 *	<0.001 *
PLTs × 10^3^/mm^3^	162.5(115.2–234.0)	94.0(60.0–149.0)	244.5(223.0–276.0)	<0.001 *	<0.001 *	<0.001 *
INR	1.2(1.1–1.40)	1.5(1.2–1.8)	1.1(1.0–1.3)	<0.001 *	0.006 *	<0.001 *
Lymphocyte %	25.1(21.8–32.4)	21.0(15.0–30.0)	23.5(20.6–29.7)	0.032 *	NS	NS
PLR	92.8(58.3–145.7)	62.4(38.2–94.8)	117.7(93.1–172.0)	0.004 *	0.072	<0.001 *
AFP (ng/mL)	101.7(17.6–386.2)	6.0(3.3–10.2)	4.5(2.8–6.6)	<0.001 *	<0.001 *	NS
Tc%	19.4(13.4–25.7)	17.0(13.7–20.0)	17.4(11.9–28.0)	NS	NS	NS
LAIR-1 MFI on Tc	39.8(31.0–51.1)	32.0(26.0–42.0)	22.0(17.5–31.2)	0.012 *	<0.001 *	<0.001 *
LAIR-1+Tc %	82.8(64.7–90.0)	65.0(38.5–77.4)	13.6(8.28–19.0)	<0.001 *	<0.001 *	<0.001 *
GLR	34.4(21.4–56.9)	27.8(14.8–39.7)	9.1(7.6–11.0)	NS	<0.001*	<0.001 *

Data are median(interquartile range (1st–3rd quartile)), statistics were computed using SPSS software, Mann–Whitney test was used (non-parametric data), *p1* indicates comparison between number of populations in HCV G4-related HCC & HCV G4-related liver cirrhosis groups, *p2* indicates comparison between number of populations in HCV G4-related HCC & healthy control, *p3* denotes comparison between number of populations in HCV G4-related liver cirrhosis & healthy control, * statistical significance *p*-value < 0.05, NS, non-significant. [ALT, alanine aminotransferase; AST, aspartate aminotransferase, ALP, alkaline phosphatase; AFP, alpha feto protein, BMI, Body mass index; DM, diabetes, GGT, gamma glutamyl transferase; GLR, GGT-to-lymphocytes ratio, Hgb; hemoglobin; HDL, high-density lipoprotein; INR, international normalized ratio; LAIR-1, leukocyte-associated immunoglobulin-like receptor-1, leukocyte-associated immunoglobulin-like receptor-1 LC, liver cirrhosis; PLT, platelet; PLR, platelet/lymphocyte ratio; TAG, triacylglycerol; Tc. T cytotoxic; TLC, total leukocytic count.].

**Table 3:** Pathological characteristics of the studied HCV G4-related HCC cases (*n* = 64) and HCV G4-related liver cirrhosis cases (*n* = 37).

Groups, *n*(%)	HCC, 64 (100%)	Liver Cirrhosis, 37(100%)	Statistics Test, *p*-Value
	Pathological characteristics		
Parameters	Ascites		*X^2^* = 9.0, 0.03 *
No	43 (67.2%)	18 (48.6%)	
Minimal	9 (14.1%)	2 (5.4%)	
Moderate	8 (12.5%)	12 (32.4%)	
Marked or Massive	4 (6.3%)	5 (13.5%)	
	Lung radiological findings		*X^2^* = 0.48, 0.48
Normal	57 (89.1%)	35 (94.6%)	
Abnormal findings #	7 (10.9%)	2 (5.4%)	
BCLC staging			N.A
0 very early	0 (0%)	-	
A early	18 (28.1%)	-	
B intermediate	16 (25.0%)	-	
C advanced	19 (29.7%)	-	
D terminal	11 (17.2%)	-	
CHILD score for liver disease severity			
A least severe	36 (56.3%)	11 (29.7%)	*X^2^* = 11.7, 0.003 *
B moderately severe	18 (28.1%)	9 (24.3)	
C most severe	10 (15.6%)	17 (45.9%)	
	LN involvement		*X^2^* = 6.4, 0.012 *
N0	54 (84.4%)	37 (100.0%)	
N1/Yes	10 (15.6%)	0 (0.0%)	
Liver size ^$^	16.3 ± 2.8	12.7 ± 2.0	*t*-test = 7, <0.001
Splenomegaly ^$^	16.5 ± 2.1	17.6 ± 4.4	*t*-test = 1.7, 0.8
Portal vein^dilatation up to 13	51.33 (3285)	50.43 (1866)	U-test = 1163, 0.88
	Liver pattern		N.A
Heterogenous mass	3 (4.7%)	0 (0.0%)	
Focal single lesion	40 (62.5%)	0 (0.0%)	
Multiple lesions	21 (32.8%)	0 (0.0%)	
Cirrhotic	0 (0.0%)	37 (100.0%)	
	Liver mass number		N.A
1.00	32 (50.0%)	0 (0.0%)	
2.00	7 (10.9%)	0 (0.0%)	
3.00	2 (3.1%)	0 (0.0%)	
≥4.00	23 (35.9%)	0 (0.0%)	
	Portal vein patency		N.A
Patent	45 (70.3%)	37 (100.0%)	
Partially occluded	4 (6.3%)	0 (0.0%)	
Thrombosed	15 (23.4%)	0 (0.0%)	

^$^ Data shown as mean ± S.D for parametric data or ^ or *n* frequencies, % for non-parametric data, statistics were computed using SPSS software, using Student’s *t*-test (parametric data), Chi square test *X^2^* (dichotomous parameters), or Mann–Whitney U-test (non-parametric data), * statistically significant at *p* < 0.05, # 2 patients have effusion, 4 patients have nodules, 1 collapse, 1 consolidation. N.A.; not applicable/not available.

**Table 4:** Expression levels of LAIR-1+Tc % and LAIR-1 MFI on Tc in all cases (*n* = 101) stratified subgroups, when the cutoff values of the demographic or the clinicopathological parameters estimated/measured were considered.

Group *n*, % Characteristics	Patients, 101, 100%		LAIR-1	
	N	%	MFI on Tc	+Tc%
Gender				
Male	78	77.3	38.75 (29.85–49.10)	75.62 (60.00–86.25)
Female	23	22.7	38.00 (26.90–54.00)	74.20 (45.5–90.50)
*p*-value			NS	NS
Age (years)				
<60	41	40.6	38.00 (30.25–56.00)	76.00 (60.74–88.50)
≥60	60	59.4	39.00 (28.93–47.28)	74.10 (56.08–85.90)
*p*-value			NS	NS
BMI (kg/m^2^)				
Under-weight > 25	8	7.9	41.50 (30.75–61.43)	76.00 (51.05–88.13)
Lean 25–29.9	45	44.6	37.00 (29.20–48.50)	75.23 (62.74–88.00)
Over-weight 30–34.9	37	36.7	40.60 (30.62–56.00)	84.00 (62.00–87.48)
Obese 35–39.9	5	4.9	29.00 (23.73–39.50)	56.07 (48.97–80.23)
Morbid-obesity ≥ 40	6	5.9	32.00 (22.75–33.35)	46.55 (35.38–66.45)
*p*-value			NS	NS
Diabetes Mellitus				
Yes	43	42.6	39.00 (28.9–50.00)	73.20 (56.00–86.00)
No	58	57.4	37.50 (30.23–49.10)	77.00 (61.20–87.81)
*p*-value			NS	NS
Insulin resistance				
Yes	62	61.4	40.30 (30.38–55.25)	82.80 (59.7–89.25)
No	39	38.6	33.00 (28.00–40.00)	73.2 (56.55–82.60)
*p*-value			0.007	0.038
AFP (ng/mL)				
<20	48	47.5	34.00 (28.68–43.05)	70.69 (55.55–84.00)
≥20	53	52.5	40.00 (31.02–55.00)	83.00 (69.66–90.00)
*p*-value			0.023	<0.001
s.Albumin (gm/dL)				
<2.5	20	19.8	31.35 (24.25–40.25)	73.60 (43.40–82.00)
≥2.5	81	58.2	39.40 (30.62–50.70)	77.00 (60.19–87.60)
*p*-value			0.013	0.05
AST (U/L)				
<40	24	23.8	33.50 (25.00–46.28)	65.80 (41.25–80.50)
≥40	77	76.2	39.02 (30.37–50.70)	77.00 (60.92–88.13)
*p*-value			NS	0.010
ALT (U/L)				
<40	49	48.5	38.00 (30.85–47.85)	76.00 (51.70–88.50)
≥40	52	51.5	39.20 (28.52–52.60)	74.88 (60.00–85.98)
*p*-value			0.000	0.013
Total bilirubin (mg/dL)				
<1.4	41	40.6	38.50 (29.82–50.70)	74.00 (60.92–85.95)
≥1.4	60	59.4	38.90 (29.05–46.00)	75.62 (56.00–87.61)
*p*-value			NS	NS
Direct bilirubin (mg/dL)				
≤0.4	21	20.8	34.00 (28.65–43.55)	62.80 (52.74–79.23)
>0.4	80	79.2	39.50 (30.29–51.05)	76.50 (61.00–87.94)
*p*-value			0.0182	0.048
ALP (U/L)				
<145	27	26.7	37.09 (28.77–49.55)	74.00 (56.05–85.90)
≥145	74	73.3	40.00 (32.00–49.00)	83.02 (73.00–88.50)
*p*-value			NS	0.042
GGT (U/L)				
<40	25	24.8	33.00 (28.01–47.00)	66.60 (46.55–82.00)
≥40	76	75.2	39.20 (30.06–49.85)	77.20 (60.09–87.94)
*p*-value			NS	0.040
Total cholesterol (mg/dL)				
<200	79	78.2	38.00 (29.00–47.70)	74.20 (57.65–85.90)
≥200	22	21.8	39.80 (29.35–56.00)	82.80 (59.61–87.90)
*p*-value			NS	NS
TAG (mg/dL)				
<150	64	63.4	37.59 (28.52–45.25)	74.26 (56.02–87.75)
≥150	37	36.6	40.02 (30.37–53.00)	77.01 (64.00–86.50)
*p*-value			NS	NS
HDL-C mg/Dl				
<35	53	52.5	35.00 (29.20–42.50)	74.00 (57.98–85.90)
≥35	48	47.5	40.50 (29.65–52.75)	78.00 (59.47–87.15)
*p*-value			NS	NS
TAG/HDL-C ratio				
<2.4	25	24.8	33.00 (26.29–39.70)	64.01 (40.00–80.03)
≥2.4	76	75.2	39.80 (30.78–52.00)	77.43 (61.28–87.94)
*p*-value			0.009	0.011
TLC × 10^3^/mm^3^				
<4.5	24	23.8	35.50 (29.55–47.80)	74.00 (60.70–82.60)
≥4.5	77	76.2	39.00 (29.00–49.50)	77.40 (56.04–88.75)
*p*-value			NS	0.0228
PLT × 10^3^/mm^3^				
<150	57	56.4	35.00 (28.50–48.00)	75.23 (57.55–86.45)
≥150	44	43.6	39.00 (30.42–49.30)	75.26 (59.91–87.75)
*p*-value			NS	NS
INR				
<1.1	11	10.9	38.20 (29.20–56.00)	90.00 (59.88–91.00)
≥1.1	90	89.1	38.75 (29.30–49.1)	74.87 (58.51–85.90)
*p*-value			NS	NS
PLR				
<81.4	50	49.5	33.50 (28.01–43.05)	72.50 (52.85–87.05)
≥81.4	51	50.5	40.03 (32.00–51.40)	78.00 (62.80–87.25)
*p*-value			0.023	NS
GLR				
<20.5	30	29.7	32.00 (27.15–42.25)	64.50 (44.63–85.23)
≥20.5	71	70.3	39.60 (30.50–39.60)	77.00 (64.00–87.75)
*p*-value			0.030	0.040
Ascites				
0, No	61	60.4	38.20 (28.95–49.20)	74.00 (59.94–86.45)
1, Minimal	11	10.9	46.00 (40.00–58.00)	84.90 (76.00–88.50)
2, Moderate	20	19.8	38.50 (29.38–45.30)	75.00 (48.13–88.90)
3, Marked	9	8.9	32.00 (26.00–30.09)	55.40 (41.80–88.38)
*p*-value			0.027 (Marked vs. Minimal, *p* = 0.020)	NS
BCLC staging (*n* = 64)				
0 very early	0	0		
A early	18	28.1	39.20 (29.75–44.18)	86.45 (60.00–90.20)
B intermediate	16	25.0	47.50 (32.30–51.85)	78.30 (65.42–85.82)
C advanced	19	29.7	40.00 (31.00–55.00)	83.00 (71.00–90.50)
D terminal	11	17.2	37.18 (32.00–54.00)	84.70 (77.00–89.00)
*p*-value			NS	NS
Child score for liver disease severity				
A least severe	47	46.6	35.00 (28.90–48.00)	74.00 (60.00–90.00)
B moderately severe	27	26.7	40.60 (37.00–50.00)	82.60 (65.00–86.00)
C most severe	27	26.7	32.00 (29.00–50.00)	74.52 (45.50–87.20)
*p*-value			NS	NS
LN				
No/Yes	10	9.9	34.50 (28.15–48.25)	73.50 (56.21–88.73)
No	91	90.1	39.00 (29.40–49.40)	75.23 (59.88–787.20)
*p*-value			NS	NS
Liver pattern				
Heterogenous mass	3	3.0	50.00 (31.00-NA)	77.00 (73.00-NA)
Focal single lesion	40	39.6	39.70 (29.40–49.30)	83.00 (62.10–90.00)
Multiple lesions	21	20.8	39.60 (34.00–54.00)	79.00 (66.59–88.38)
Cirrhotic	37	36.6	32.00 (26.00–42.00)	65.00 (38.50–77.43)
*p*-value			NS	*<0.001* (Cirrhotic vs focal, *p* < 0.001) (Cirrhotic vs multiple, *p* = 0.018)
Liver mass number (*n* = 64)				
1	32	50.0	40.45 (30.42–50.00)	83.65 (60.00–90.08)
2	7	10.9	28.90 (26.90–46.00)	69.66 (62.8–83.00)
3	2	3.1	37.14 (22.88–NA)	69.28 (55.93–NA)
≥4	23	36.0	40.00 (36.00–56.00)	84.90 (74.20–90.50)
*p*-value			NS	NS
Lung findings				
Abnormal	9	8.9	33.00 (31.50–44.50)	79.00 (55.70–87.75)
Normal	92	91.1	38.75 (29.05–49.85)	74.36 (59.91–86.75)
*p*-value			NS	NS
Portal vein patency				
Patent	82	81.2	38.35 (28.98–49.55)	73.10 (56.05–85.42)
Partially occluded	4	3.9	35.70 (32.00–40.30)	82.10 (60.10–90.75)
Thrombosed	15	14.9	40.00 (32.00–54.00)	84.90 (76.00–90.00)
*p*-value			NS	0.018 (Patent vs thrombosed, *p* = 0.018)

All data were expressed as median (quartiles) and comparison was assessed using Mann–Whitney test (U) for comparison of two non-parametric groups and Kruskal–Wallis one-way ANOVA (H) for more than two non-parametric groups on SPSS software, NS; none-significant. [ALT, alanine aminotransferase; AST, aspartate aminotransferase, ALP, alkaline phosphatase; AFP, alpha feto protein, BMI, Body mass index; DM, diabetes, GGT, gamma glutamyl transferase; GLR, GGT-to-lymphocytes ratio; Hgb; hemoglobin; HDL, high-density lipoprotein; INR, international normalized ratio; LAIR-1, leukocyte-associated immunoglobulin-like receptor-1, leukocyte-associated immunoglobulin-like receptor-1 LC, liver cirrhosis; PLT, platelet; PLR, platelet/lymphocyte ratio; TAG, triacylglycerol; Tc. T cytotoxic; TLC, total leukocytic count.].

**Table 5:** Spearman’s correlation coefficient among investigated LAIR-1 expressions LAIR-1+Tc% and LAIR-1 MFI on Tc cells in all patients (*n* = 101).

	Patients (*n* = 101)			
	LIAR-1 MFI on Tc		LIAR-1+Tc%	
Characteristics	*r*	*p*-Value	*r*	*p*-Value
Age (years)	−0.063	NS	−0.063	NS
BMI (kg/m^2^)	−0.112	NS	−0.155	NS
s.Insulin (mIU/L)	0.035	NS	0.044	NS
AFP (ng/mL)	0.218	0.028 *	0.213	<0.001 *
AST (U/L)	0.104	NS	0.178	NS
ALT (U/L)	0.038	NS	0.101	NS
ALP (U/L)	0.115	NS	0.096	NS
GGT (U/L)	0.106	NS	0.176	NS
TAG (mg/dL)	0.125	NS	0.127	NS
Total Cholesterol (mg/dL)	0.134	NS	0.178	NS
HDL-C (mg/dL)	0.061	NS	0.055	NS
TAG/HDL-C	0.111	NS	0.077	NS
TLC × 10^3^ mm^3^	0.124	NS	0.076	NS
PLT × 10^3^ mm^3^	0.101	NS	0.092	NS
PLR	0.108	NS	0.053	NS
GLR	0.196	0.049 *	0.178	NS
Liver size	0.190	NS	0.401	<0.001 *
Insulin resistance #	0.302	0.002 *	0.188	NS
BCLC staging #	−0.037	NS	0.141	NS
Child score for liver disease severity #	−0.045	NS	−0.117	NS
Portal vein patency #	0.050	NS	0.271	0.006 *

Spearman correlation coefficient (*r*) was calculated using SPSS software, * significant correlation at *p* < 0.05 level (2-tailed), NS; none significant, # analyzed by point-biserial correlation. [ALT, alanine aminotransferase; AST, aspartate aminotransferase, ALP, alkaline phosphatase; AFP, alpha feto protein, BMI, Body mass index; DM, diabetes, GGT, gamma glutamyl transferase; GLR, GGT-to-lymphocytes ratio, Hgb; hemoglobin; HDL, high-density lipoprotein; INR, international normalized ratio; LAIR-1, leukocyte-associated immunoglobulin-like receptor-1, leukocyte-associated immunoglobulin-like receptor-1 LC, liver cirrhosis; PLT, platelet; PLR, platelet/lymphocyte ratio; TAG, triacylglycerol; Tc, T cytotoxic; TLC, total leukocytic count.].

**Table 6:** ROC curve for the discriminative ability of LAIR-1+Tc% and LAIR-1 MFI on Tc to differentiate HCC from liver cirrhosis.

		%					Asymptotic 95% CI	
Variables	Cut-Off Point	Sensitivity	Specificity	AUC	S.E.	*p*-Value	Lower Bound	Upper Bound
LAIR-1+Tc%	73.6	67.2	62.2	0.756	0.049	<0.001 *	0.661	0.851
LAIR-1 MFI on Tc	34.5	67.2	62.2	0.651	0.058	0.012 *	0.538	0.764
AFP (ng/mL)	12.5	81.2	89.2	0.876	0.035	<0.001 *	0.807	0.946
LAIR-1+Tc% + LAIR-1 MFI on Tc	--	20	60	0.753	0.049	<0.001 *	0.656	0.850
LAIR-1+Tc% + AFP	--	83	70	0.912	0.027	<0.001 *	0.858	0.965
LAIR-1 MFI on Tc + AFP	--	82	65	0.885	0.033	<0.001 *	0.821	0.949
LAIR-1+Tc% + LAIR-1 MFI on Tc + AFP	--	85	73	0.918	0.027	<0.001 *	0.865	0.970

Data calculated using SPSS software, * significant correlation at *p* < 0.05 level (2-tailed). [AFP, alpha feto protein; LAIR-1, leukocyte-associated immunoglobulin-like receptor-1, MFI, mean fluorescence intensity, Tc, T cytotoxic.].

## Data Availability

The original contributions presented in the study are included in the manuscript. Further inquiries can be directed to the corresponding author.
